# The Relationship between Age at First Birth and Mother's Lifetime Earnings: Evidence from Danish Data

**DOI:** 10.1371/journal.pone.0146989

**Published:** 2016-01-22

**Authors:** Man Yee Mallory Leung, Fane Groes, Raul Santaeulalia-Llopis

**Affiliations:** 1 Division of Public Health, Department of Surgery, Washington University in St. Louis, St. Louis, Missouri, United States of America; 2 Department of Economics, Copenhagen Business School, Copenhagen, Denmark; 3 Department of Economics, Washington University in St. Louis, St. Louis, Missouri, United States of America; Iowa State University, UNITED STATES

## Abstract

**Background:**

Having children creates career interruptions and reductions in labor income for women. This study documents the relation between the age at first birth (AFB) and women’s labor income. We study these dynamics in the short run (i.e. ratio between labor income at AFB and two years prior to AFB) and long run (i.e., positive/negative differences in total lifetime labor income).

**Methods:**

Using unique Danish administrative register data for the entire Danish population, we estimate the age-income profiles separately for college and non-college women conditional on marital status, and mothers’ age at first birth (AFB). We compute the lifetime labor income differentials by taking the differences between the labor income of women with and without children at each AFB.

**Results:**

The short-run loss in labor income, defined as the difference in percentages between the income earned two years prior to AFB and income earned at AFB, ranges from 37% to 65% for college women and from 40% to 53% for non-college women. These losses decrease monotonically with respect to AFB for both education groups. Our results on the lifetime labor income differentials between mothers and women without children also show a net effect that is monotonic (from negative to positive) in AFB. With AFB<25, the lifetime labor income loss for college women is -204% of their average annual labor income and this figure is -252% for non-college women. There are lifetime labor income gains with AFB>31. The largest gains for college women are 13% of their average annual income and this figure is 50% for non-college women.

**Conclusion:**

Women have a large and unambiguous short-run reduction in labor income at their AFB. In terms of lifetime labor income, both college and non-college women, compared to childless women, are associated with lower income of more than twice their respective average annual income when bearing a child at AFB<25. In other words, women with AFB<25 are associated with a lower lifetime income of more than two years of annual labor income. The lifetime labor incomes for college and non-college women associated with AFB>31 are relatively higher.

## Introduction

It has been widely documented that mothers who work full time in a paid job typically earn less income than women who never have children [1]. This gap is known as the “motherhood gap” or “family gap”. Here, we estimate this gap using administrative panel data for the entire Danish population from 1995 to 2009. The length, size and richness of our panel data allow us to study how much the entire lifetime labor income of college and non-college women is related with differentials in the mother’s age at delivery.

Several hypotheses have been proposed to explain the existence of the motherhood gap. First, mothers are more likely to be interrupted in their careers due to maternity leave. This leads to depreciation in human capital which results in a lower return in the labor market [1–4]. Second, mothers self-select into the family-friendly, but lower wage, public sector, resulting in lower labor market returns to women with children compared with women without any children [5] or experience a lower pay because they spend more time in activities that do not involve human capital accumulation such as work or school [6]. Third, women with family ties usually face constraints and frictions in the job search, implying lower job mobility compared with women without family ties [7]. Fourth, there is unobserved heterogeneity such as “career motivation” among women with different abilities and characteristics that may be correlated with their fertility choice [2]. Notice that these are primarily are hypotheses related to the motherhood gap, since results show that fathers, contrary to mothers, enjoys a fatherhood premium in the labor market [8,9].

The majority of the literature estimating the impact of pregnancy has focused on wages to understand the determinants of the motherhood gap [1,4,10–14].For example, Ejrnaes and Kunze used the Register data on West Germany and found that a mother’s wages drop by 3 to 5.7% per year of maternity leave [10]. Datta Gupta and Smith used the 5% panel data set of the Danish population of the years 1980–1995 to analyze the effects of children on women’s wages. They found that the family gap disappeared when unobserved heterogeneity and self-selectivity were controlled [4]. Budig and England find a wage penalty for motherhood of 7% per child among young American women with about one-third of the penalty being explained by years of past job experience and seniority, including part-time past work [12]. While these studies focused on the effect of children on women’s wages, here instead, we focus on total labor income. In that context, Phipps et al. also studied the impact of first pregnancy on women’s total labor income in Canada for a sample of 1,296 women [1]. Their results suggest that women who ever had a child reduced their labor income by 17.2%. However, when time out of the labor force was controlled for, their estimate of income penalty fell to 12.5%. Due to limitations in sample size and/or characteristics, this literature has been confined to the measurement of the size of income penalty without consideration of the timing and age at first pregnancy. Here, we fully incorporate in our estimation the timing of age at first birth (AFB) as potential determinants of the motherhood gap.

More recently, Miller and Karimi pursue causality with the use of miscarriages as instruments to control for the selection of timing of the first child. Miller finds that delaying childbirth by one year leads to a 9% increase in earnings from age 21 to 34 in the US, a medium-run effect on earnings [11], while Karimi, applying the same instrument to Swedish data, finds that one-year delay implies a reduction on earnings [15]. Besides estimating causal effects of AFB, Miller and Karimi provide OLS estimates, as we do, that suggest that delays in AFB are associated with medium-run increases in earnings [11,15]. Instead, in our exercise, we are able to distinguish short-run and lifetime labor income differences associated with AFB.

The aim of this paper is to document the joint dynamics between of age at first pregnancy and the subsequent women’s labor income in the short run and through the entire lifetime without establishing causal effects. To this end, we use the Danish administrative register data, which allows us to compute labor income of women with different ages at first birth and keep track of them for fifteen years. Uncovering the age-income profile by exploiting cohort variation, as we do, enables us to study how AFB relates to women’s short-run and lifetime labor income.

## Materials and Methods

### Data

We use the Danish administrative register data, which is a panel dataset covering 100% of the population in the years 1995 to 2009. The data is provided by Statistics Denmark [16] and includes many different registers. We use registers with annual information on socioeconomic variables (e.g., age, gender, education, etc.), income information (yearly income, earnings and a crude measure of wealth), characteristics of employment (e.g., employed, self-employed, unemployed etc.), and family links (link to identification of spouse/cohabiting partner, children, parents, and other household members) of the population. Women in the sample are linked with their children through family links and personal identification numbers. The exclusion criteria were as follows: (i) we focus on women who are married or cohabitating; (ii) we focus on women of ages 25 to 60, as they have most likely already completed their education; (iii) we exclude individuals who are self-employed and women who do not work in a given year because they have no earnings measure. The reason to exclude single women follows a comparability principle. That is, the predictive probabilities of having a child and the earnings of within married/cohabitating women substantially differ from singles. This criterion implies that in a given year, we have three groups of married/cohabitating women: 1) Women without children throughout the entire sample period, 2) women without children in the year under consideration but whom have children later in the sample and, 3) women who already had a child. Note that if having children has heterogeneous effects on the probability of staying married across earned income this may bias our results because of the sample selection of only married women.

We focus on the joint dynamics between the age at first birth and lifetime labor income for college and non-college women. Since our analysis focuses on the gross effect of AFB on the labor income, this effect is the product of two components: (1) the differences in wage rate and (2) differences in labor inputs between mothers and childless women in our sample. We focus on labor income because we only have a rough measure for hours in the register data. The labor income of the female population was converted in real terms to the year 2000 price level using the Danish Consumer Price Index obtained from Danish National Accounts. The AFB of a woman was obtained by subtracting the age of her oldest child from her current age. Women were then classified into 8 groups according to their AFB: no children, AFB<25, 25≤ AFB <28, 28≤ AFB<31, 31≤ AFB<34, 34≤ AFB<37, 37≤ AFB <40 and AFB ≥ 40. This classification implies that our data set covers cohorts born from 1935 to 1983.

### Descriptive Statistics

Table 1 presents the summary statistics of our sample from 1995–2009. The sample contains 1,597,805 women ranging in age from 25 to 60 with a total of 8.5 million observations. For year 1995, 23.6% of the women had a college or higher degree, and 80.0% were married. These sociodemographic characteristics have changed over time. In 2009, 34.6% of the women had a college or higher degree (an increase by almost 50% compared with 1995), and 78.3% were married. The college premium for women, which is defined as the ratio of earnings between college and non-college graduates [17], is similar across the entire period, DKK 235,458/DKK 173,682 = 1.36 in 1995 and DKK 287,093.5/DKK 217,283.6 = 1.32 in 2009. The average labor income over the sample in 2000 prices was DKK 188,253, which is equivalent to US$33,886 at the exchange rate of DKK1: US$0.18.

**Table 1:** Descriptive Statistics of Danish administrative register data (1995–2009).

**Year 1995**	**College Women**			**Non-College Women**		
	**N**	**(%)**		**N**	**(%)**	
**Age at First Birth**		**Total sample**	**AFB sample**		**Total sample**	**AFB sample**
No children	28,650	12.5	-	80,285	10.8	—
AFB<25	51,964	22.7	25.9	356,648	48.1	53.9
25≤AFB<28	57,169	25.0	28.5	145,577	19.6	22.0
28≤AFB<31	45,208	19.7	22.6	83,233	11.2	12.6
31≤AFB<34	24,932	10.9	12.4	42,258	5.7	6.4
34≤AFB<37	12,588	5.5	6.3	20,933	2.8	3.2
37≤AFB<40	5,990	2.6	3.0	9,696	1.3	1.5
AFB≥40	2,543	1.1	1.3	3,399	0.5	0.5
Total	229,044	100.0	100.0	742,029	100.0	100.0
				**Mean**	**Std Dev.**	
Fraction with College degree				23.6%	-	
Fraction of women who are married				80.0%	-	
Average labor income in 2000 prices				DKK 188,253	98,628	
Average labor income of college women				DKK 235,458	105,649	
Average labor income of non-college women				DKK 173,682	91,567	
Average age at first birth: Total/College/Non-college				25.4/27.5/24.8	4.7/4.6/4.6	
**Year 2009**	**College Women**			**Non-College women**		
	**N**	**(%)**		**N**	**(%)**	
**Age at First Birth**		**Total sample**	**AFB sample**		**Total sample**	**AFB sample**
No children	89,465	23.4	—	133,084	18.4	—
AFB<25	59,231	15.5	20.3	273,132	37.8	46.3
25≤AFB<28	78,261	20.5	26.8	147,602	20.4	25.0
28≤AFB<31	79,379	20.8	27.1	93,077	12.9	15.8
31≤AFB<34	45,139	11.8	15.4	45,831	6.3	7.8
34≤AFB<37	19,676	5.2	6.7	19,829	2.7	3.4
37≤AFB<40	7,743	2.0	2.6	8,043	1.1	1.4
AFB≥40	3,017	0.8	1.0	2,684	0.4	0.5
Total	381,911	100.0	100.0	723,282	100.0	100.0
				**Mean**	**Std Dev.**	
Fraction with College degree				34.6%	-	
Fraction of women who are married				78.3%	-	
Average labor income in 2000 prices				DKK 241,407	121,798	
Average labor income of college women				DKK 287,093	142,068	
Average labor income of non-college women				DKK 217,283	101,620	
Average age at first birth: Total/College/Non-college				26.2/28.0/25.4	4.6/4.4/4.5	

In terms of AFB, we note that, on average, women were having children almost a year later in 2009 than in 1995, respectively, 26.2 and 25.4. The college gap in AFB years remains steady around 2.6 between 1995 and 2009. We find a substantial increase between 1995 and 2009 in the proportion of women who have had no children by a factor of 23.4%/12.5% = 1.87 and 18.4%/10.8% = 1.70, respectively, for college and non-college women. Hence, the gap between childless college women and childless non-college women widened in this period by a factor of 23.4/18.4 = 1.27 in 2009 compared with 12.5/10.8 = 1.15 in 1995. We observe an increase in the proportion of college women that, conditional on having a child, deliver between ages 28 and 34, while this proportion declines for ages below 28. Regarding non-college women, we observe an increase in the proportion of non-college women that, conditional on having a child, deliver after age 25 while this proportion declines for ages below 25. Overall, there is an increase in AFB and the distributional shift toward older ABF is more pronounced in college women than in non-college women.

### Outcome Variables, Covariates, and Model Specifications

To examine the relationship between age at first birth and women's age-income profile, we use a parametric approach with a linear model specified as follows:

$$I_{it}={\beta_{0}+\pi }_{c}{cohort}_{it}+\beta_{age}{age}_{it}+\lambda_{g}^{a}{(age}_{it}*{AFB}_{g})+\gamma_{g}{AFB}_{g}+\theta_{m}{marital\;status}_{it}+\varepsilon_{it}$$

Consider individual *i* at time *t* who gave birth at some time in her life at *AFB*_*i*_ which can be classified into one of the age at first birth groups *g* ∈ {< 25, [25, 28), [28, 31), [31,34), [34, 37), [37, 40), ≥ 40}. To analyze the association between age at first birth and women’s labor income, the labor income of individual *i* at time *t* (in 2000 prices), *I*_*it*_, was regressed on a constant (*β*_0_); *cohort*_*it*_ that includes dummies for each birth cohort; *age*_*it*_ that includes a vector of 36 one-year dummy (for ages 25–60); *AFB*—a dummy for each group of age at first pregnancy; an interaction term between *age*_*it*_ and *AFB*_*g*_; and *marital status*_*it*_ which is a dummy indicating whether the individual was married at time *t*. While our data cover a large sample of women from a wide range of ages, the duration of the panel (15 years), is shorter than the range of ages (spanning 36 years) that we study. This justifies the introduction of cohort effects in our estimation equation to net our lifetime results from potential cross-cohort level differences (e.g. in education and motherhood). We find our results are robust to the introduction of year effects identified as to capture short-run variation as in [18].

**Fig 1:**
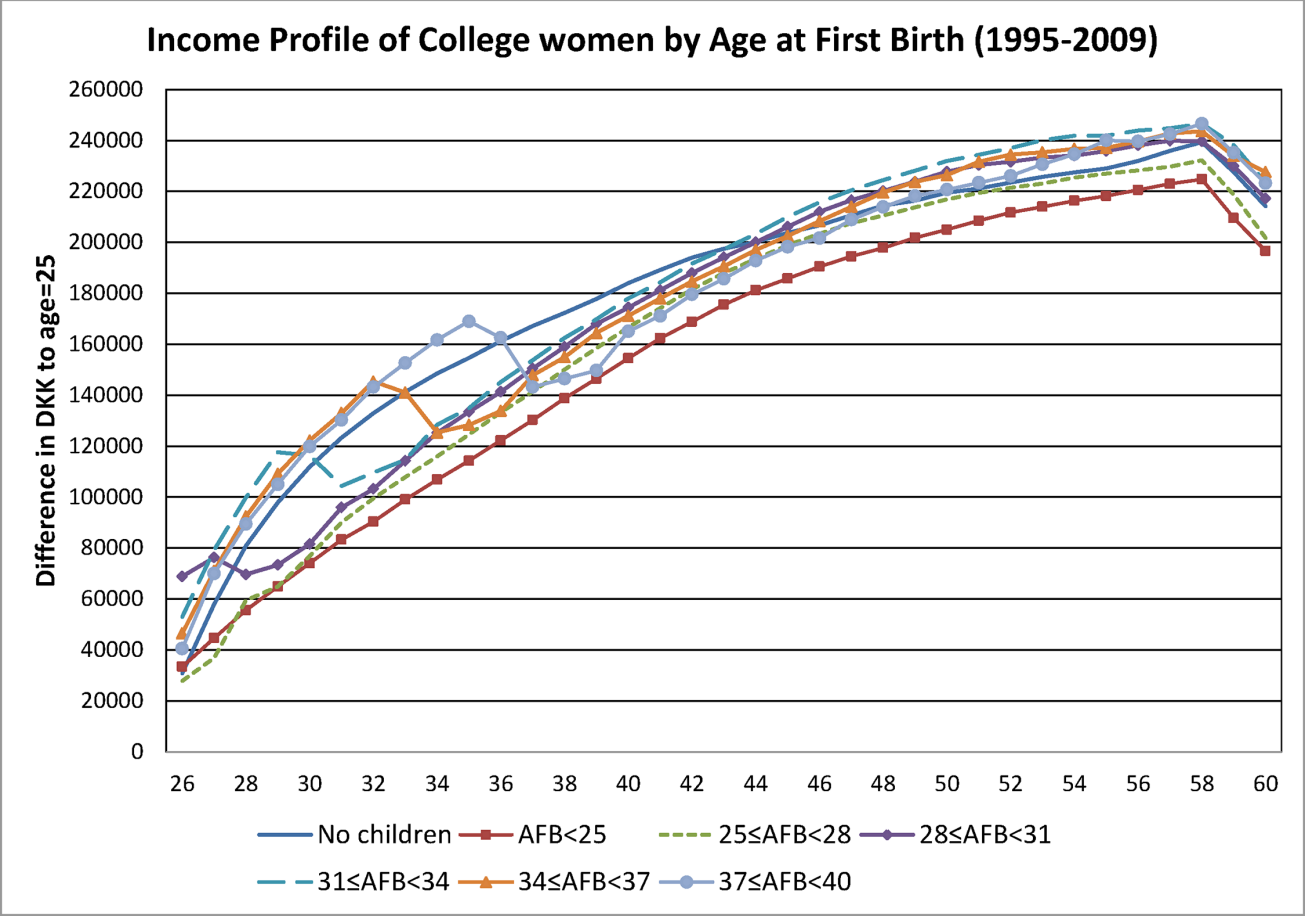
Income Profile of College Women by Age at First Birth (1995–2009).

**Fig 2:**
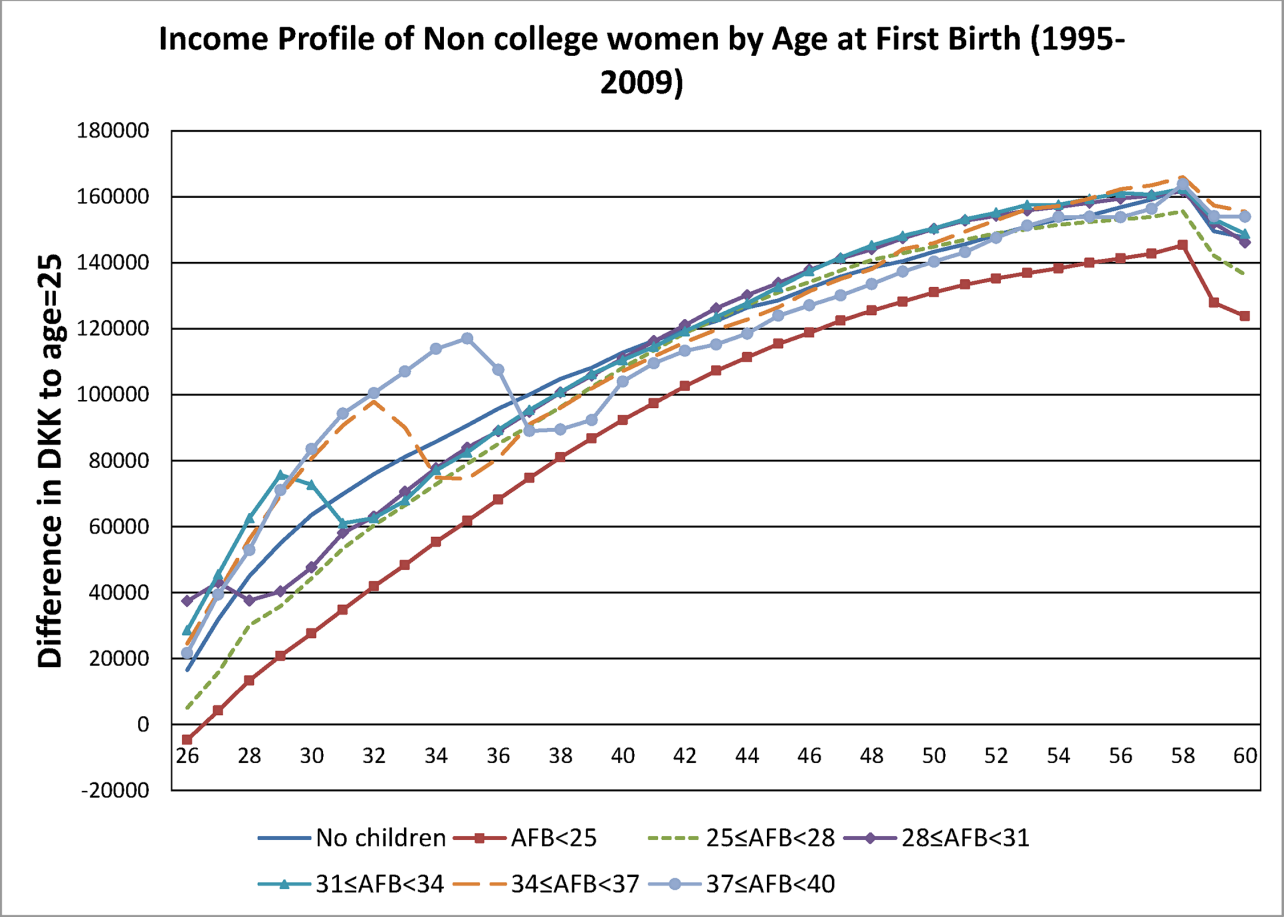
Income Profile of Non-College Women by Age at First Birth (1995–2009).

The regression model was estimated using OLS. The inclusion of *cohort*_*it*_ controlled for cohort fixed effects in the model. The coefficients on the age dummies, *β*_*age*_, represent the impact of the life cycle conditional on birth cohort and marital status for women who have no children. While the interaction term between *age*_*it*_ and *AFB*_*g*_ captures potential differences of age-income profile across different age at first birth groups. Age-labor income profiles conditional on *AFB*, i.e., the predicted values ${(\hat{\beta }}_{age}+{\hat{\lambda }}_{g}^{a}+{\hat{\gamma }}_{g})$, are plotted in Figs 1 and 2 for college and non-college women, respectively.

Given the shorter duration of the panel (15 years) compared with the age range of the lifecycle (36 years), one should note that the lifecycle labor income profile that is uncovered from the above strategy are averages over ages across women of different cohorts. For the expositional purpose of Figs 1 and 2, we choose three year intervals of AFB. However, the stratification of women into groups of AFB reduces the size of income reduction around the time when pregnancy occurs. This is because the classification of women into AFB groups averages the impact of AFB on labor income around AFB over three-year periods. To this end, we estimate the regression with both 3-year AFB groups as well as 1-year dummies of AFB. Since such reduction in magnitude is particularly stark for the SR gains/losses, we report the SR gain/loss using the 1-year AFB dummy model. Robust standard errors clustered at the individual level and 95% confidence intervals are also computed from the estimation. Due to the large sample size, the average of the standard errors are 1514 for college women and 891 for non-college women. Since the confidence intervals are narrow relative to the size of the estimates, they are excluded in Figs 1 and 2.

To compute the lifetime difference of labor income, we take the difference between labor incomes of women at each AFB group and the labor income of childless women, i.e., women that never had a child throughout their lives. A 3% annual interest rate is used as a discount rate to compute the present value of the lifetime labor income difference. Results are summarized in Table 2. We do two sensitivity analyses. First, we restrict the sample to women with completed fertility by including women who are age 40 or above in year 2009; i.e., we include only cohorts born or before 1969. Second, we restrict the sample of mothers to those who had only one child at age 40, in order to show the relation between timing of first birth and earnings while holding number of children constant.

**Table 2:** Losses/Gains of Lifetime Labor Income by Age at First Birth.

Age at First Birth	College Women (DKK)	Non-College Women (DKK)
AFB<25	-533,943 (-2.04)	-491,243 (-2.52)
25≤AFB<28	-363,786 (-1.39)	-166,318 (-0.85)
28≤AFB<31	-123,679 (-0.47)	-20,449 (-0.10)
31≤AFB<34	33,041 (0.13)	48,140 (0.25)
34≤AFB<37	4,134 (0.02)	57,149 (0.29)
37≤AFB<40	11,365 (0.04)	97,265 (0.50)

Notes: All future income was discounted at an annual interest rate of 3%. Numbers in parentheses show the size of lifetime gain/loss of labor income relative to the average annual income at 2000 prices for college and non-college women. The average annual income for college women and non-college women in the sample is DKK 262,343 and DKK 194,848 respectively.

## Results

We divide our results in labor income short-run losses and lifetime gains/losses in labor income.

### Short-Run Loss of Labor Income by Age at First Birth

To quantify the short-run loss of labor income around the age at first birth, we estimate our econometric model using one-year dummies of AFB. We then compute the ratio between income earned 2 years prior to AFB (the highest value of income earned before AFB) and the income at the year of AFB. To summarize the results, we take averages of the ratios over AFB groups of 28≤ AFB<31, 31≤ AFB<34, 34≤ AFB<37, 37≤ AFB <40. The relative size of drop in labor income is computed only for the AFB groups of 28≤ AFB<31, 31≤ AFB<34, 34≤ AFB<37, and 37≤ AFB <40. The reason is that the highest and the trough values of labor income for the AFB groups AFB<25, 25≤ AFB <28 occurred before age 25. Our results suggest that for college women in AFB ∈[28, 31) a child is associated with a short-run loss of 65%; for AFB ∈[31, 34) this loss was 43%; and this loss was 39% and 37% for women in AFB ∈[34, 37), and AFB ∈[37, 40), respectively. For non-college women, our results suggest that in AFB ∈[28, 31) a child is associated with a short-run loss of 53%; for AFB ∈[31, 34) this loss was 46%; and this loss was 44%, and 40% for women in AFB ∈[34, 37), and AFB ∈[37, 40), respectively. That is, in the cases of both college and non-college women, the short-run losses decreases monotonically with AFB.

### Differences in Lifetime Labor Income by Age at First Birth

The regression model is estimated using 3-year AFB groups and the coefficients of the age dummies are plotted in Fig 1 for college women and in Fig 2 for non-college women. For college women, individuals with AFB≤28 consistently through their lifetimes earned a lower income compared with women without children. However, women with AFB>28 earn, before their first birth, a labor income above that of women without children. Then, at the time of first birth, these women experience a drop in labor income below that earned by women without children. The declining pattern in labor income persists for a few years after their first birth. Interestingly, while this labor income recovers, they continue earning a lower labor income compared with that of women without children throughout the rest of their lifetimes. For the non-college women, the trends and patterns of the income profile followed closely that of the college women. Similar to college women, non-college women with AFB≤28 almost consistently earned a lower income compared with women without children. However, after the fall in labor income that follows delivery, the income of non-college women who gave birth after the age of 28 catches up with that of women without children—a result that differs from that obtained for college women above.

It is natural to think that a better statistic underlying decision making about whether to have a child and at which age to have a child is lifetime labor income, rather than simply considering the short-run losses associated with the immediate periods that follow delivery. Table 2 shows the lifetime losses/gains in labor income expressed, respectively, as negative/positive differences in the present value of labor income by AFB groups separately for college and non-college women:

$$\sum_{a}^{A}\frac{{\hat{\lambda }}_{g}^{a}+{\hat{\gamma }}_{g}}{{\left(1+r\right)}^{a-25}}$$

where ${\hat{\lambda }}_{g}^{a}+{\hat{\gamma }}_{g}$ is the difference between the labor income of women in AFB group *g* and the labor income of women without children at each age *a*. The difference in lifetime income is also calculated using the regression model with yearly dummies of AFB. This difference is computed for each AFB and averaged over the 3-year AFB groups. To compute lifetime losses/gains, we discount the labor income path at the interest rate *r* = 3%. For college women the difference in total lifetime labor income compared with women without children is negative if AFB<31, and the difference is positive if AFB>31. For women with AFB<31 the loss in lifetime labor income declines with AFB. The loss in total lifetime labor income is DKK 533,943 for women with AFB<25, DKK 363,786 for women with AFB ∈[25, 28); and DKK 123,679 for women with AFB ∈[28, 31). Women with AFB≥31 have higher lifetime labor income compared with women without children. The gain is DKK 33,041 for women with AFB ∈[31, 34); DKK 4,134 for women with AFB ∈[34, 37); and DKK 11,365 for women with AFB∈[37, 40). In relative terms, the lower/higher total lifetime labor income for college women relative to their average annual income over the entire sample is -204% for women with AFB<25. That is, college women who have a child before the age of 25 experience a relative lower lifetime labor income of more than two years their average. The relative loss in lifetime labor income is -139% for AFB ∈[25, 28) and -47% for AFB ∈[28, 31). For college women we find that having a first child after the age of 31 is associated with a relative higher total lifetime labor income of 13% for AFB ∈[31, 34); 2% for AFB ∈[34, 37); and 4% for AFB ∈[37, 40).

Similar to college women, non-college women with AFB<31 experience a loss of lifetime labor income. The loss for women with AFB<25 is DKK 491,243; DKK 166,318 for women with AFB ∈[25, 28); and DKK 20,449 for women with AFB ∈[28, 31). That is, the youngest mothers experience the largest lifetime labor income loss in the years that follow delivery. For women with AFB ≥ 31, non-college women experience higher total lifetime labor income. In absolute terms, the gain is DKK 48,140 for AFB ∈[31, 34); DKK 57,149 for AFB ∈[34, 37); and DKK 97,265 for AFB ∈[37, 40). Relative to their average annual income over the entire sample we find a -252% loss for AFB<25; -85% for AFB ∈[25, 28); and -10% loss for AFB ∈[28, 31). For women with AFB ≥31 the relative gains associated with AFB ∈[31, 34) are 25%; 29% for AFB ∈[34, 37); and 50% for AFB ∈[37, 40).

The results from the sensitivity analysis shown in Table 3 suggest that our findings are not sensitive to the sample selection of women with completed fertility. In Table 4, we perform a second sensitivity analysis that controls for number of children by looking only at women with one total child. When we select the sample of women with just one child, the qualitative patterns of the college and non-college women remains the same. That is, the later in life a college or non-college woman has her first (and only) child the higher is the associated lifetime earnings.

**Table 3:** Losses/Gains of Lifetime Labor Income by Age at First Birth: Sensitivity Analysis using Women with Completed Fertility.

Age at First Birth	College Women (DKK)	Non-College Women (DKK)
AFB<25	-530,474 (-2.02)	-435,941 (-2.24)
25≤AFB<28	-337,984 (-1.29)	-108,601 (-0.56)
28≤AFB<31	-117,444 (-0.45)	4,693 (0.02)
31≤AFB<34	55,703 (0.21)	88,814 (0.46)
34≤AFB<37	31,755 (0.12)	93,210 (0.48)
37≤AFB<40	49,047 (0.19)	121,582 (0.62)

Notes: Sensitivity analysis is done on women with completed fertility, i.e., women who are at least age 40 in year 2009. See also notes of Table 2.

**Table 4:** Losses/Gains of Lifetime Labor Income by Age at First Birth: Sensitivity Analysis Using Women with Completed Fertility and Only One Child or Childless.

Age at First Birth	College Women (DKK)	Non-College Women (DKK)
AFB<25	-458,432 (-1.75)	-284,438 (-1.46)
25≤AFB<28	-351,271 (-1.34)	-81,527 (-0.42)
28≤AFB<31	-201,135 (-0.77)	7,795 (0.04)
31≤AFB<34	-52,842 (-0.20)	49,216 (0.25)
34≤AFB<37	7,581 (0.03)	69,894 (0.36)
37≤AFB<40	27,057 (0.10)	138,560 (0.71)

Notes: See notes of Table 2.

## Discussion

We investigate the relationship between AFB and lifetime labor income for college and non-college women. Using data from the Danish administrative data covering 100% of the population in the years 1995 to 2009 from the Statistics of Denmark, we show that women experience a short-run drop in their total labor income after first birth that differs by AFB and ranges from 37% to 65% of the labor income earned two years before their AFB for college women and from 40% to 53% for non-college women. We note that the magnitude of the short-run drop of labor income is larger for non-college women than college women for most AFB groups except for AFB ∈[28, 31). One way to explain this difference is by considering in the replacement costs that a firm faces when replacing a worker with high versus low skills. It is easier for employers to find a substitute for a low-skilled worker compared with a high-skilled worker [19]. A second explanation could be that college women (perhaps except from the youngest group) have a stronger attachment to the labor force than non-college women, and they therefore would be more likely to have a first part of their maternity leave subsidy that is closer to their previous wages. The non-college women with lower labor force attachment often collect a maternity leave subsidy that is lower than their previous wage, and this group would therefore experience a larger drop in earnings even if they were on maternity leave.

For both college and non-college women, we find lower lifetime labor income for AFB< 31, and slightly higher lifetime labor income for AFB greater or equal to 31. These differences in lifetime labor income range from a low of -204% (with AFB<25) to a high of 13% (with AFB between 31 and 34) of average annual labor income for college women, with a largest absolute difference of DKK 533,943 with AFB<25. The differences in lifetime labor income for non-college women range from a low of -252% (with AFB<25) to a high of 50% (with AFB between 37 and 40) of average annual labor income, with the largest absolute loss of DKK 491,243 with AFB<25. Our results above are robust to variations in sample restrictions as suggested by our sensitivity analysis. The largest lifetime child loss was experienced by both college and non-college mothers with children before age 25. This result is consistent with theories of human capital accumulation that young mothers do not get to invest in as much human capital early in life as non-mothers and therefore lag behind in earnings every year after their child is born. This explains why college mothers face the largest absolute lifetime earnings difference associated with low AFB as they have the largest potential for human capital depreciation [3].

The result that college women have their largest reductions in labor income when having children early in life and the result that having the first child later in life is associated with higher earnings for women of all ages and education groups are results qualitatively similar to what Miller found in her descriptive OLS regressions, using NLSY79 data for the U.S [11]. Although the focus of Miller [11] is to estimate a causal effect of age at first birth on mother’s earnings, she also reports results from OLS regressions which are conceptually similar to our analysis and which we therefore use for comparison with our results. A differential aspect of our analysis is that we allow for a more flexible form in the AFB by including a categorical variable for the different ages at which women have their first child. While our more flexible model suggests the presence of a nonlinear trend in AFB, we find that there are diminishing returns in lifetime income with respect to AFB. The largest gain in lifetime income is associated with the first 3 years of delayed childbirth. These gains decrease as women have their first births later in life. Eventually, the differences in labor income become small or even turn negative as AFB increases. For instance, increasing AFB three years from 25–27 to 28–30 for college women gives an estimated increase in lifetime earnings of 5.4% whereas increasing AFB three years from 34–37 to 37–40 gives an increase of 0.8%.

However, one should note that mothers with children before age 25 also include teen mothers. That is, some of the mothers with a first child before age 25 belong to a highly selected group who most likely suffer from other negatively related labor market outcome shocks. Just as the group of women with a first child before age 25, the other AFB groups can also experience selection. Using miscarriages to control for selection, Miller finds that the pooled estimated effect of delayed child birth is a bit smaller in size for the IV than for the OLS, decreasing from 9.6% using the OLS to 8.8% using an IV approach [11]. In contrast, Karimi finds that, using OLS, a one-year delay of motherhood is associated with an increase in career earnings of about 3.7%, but that using the IV approach, a one-year delay of motherhood decreases earnings by 15% [15]. In this paper, we report the correlation between AFB and women labor income. Finding a way to control for the endogeneity of age at first childbirth in the Danish data could be a future extension of our results.

Our paper has several strengths: First, we have documented differences in the lifetime labor income of women associated with AFB. With the advantage of the huge database from the Danish administrative register data that obtains information of 1,597,805 women of age 25 to 60 from year 1995 through 2009, we are able to uncover the income profile of women by AFB over the life cycle. Second, our large and rich data set allows us to examine the life cycle income profile of women from age 25–60 with detailed stratification by age at first birth ∈ {no children, <25, [25, 28), [28, 31), [31, 34), [34, 37), [37, 40)}. Third, uncovering age-income paths of women by AFB enables us to measure the associated difference of both the short-run and lifetime total labor income of women with their AFB, which was not reported in previous studies.

This paper documents the association between age at first birth and women’s labor income profile for college and non-college women. The short-run drop in total labor income can be explained by factors like (i) decrease in labor productivity of mothers due to childbearing and caretaking of the newborn, (ii) labor market discrimination by employers against women with children, and (iii) lower job mobility due to family ties etc. The impact of the above stated factors on the short-run decline in mother’s labor income should be tested in future research. Our results also suggest a relationship between the timing of pregnancy and human capital accumulation in the labor market that needs further investigation for a causal interpretation. The younger a mother is, the more she has to forgo in terms, of opportunity to accumulate experience and human capital in the labor market. The younger mothers may also self-select into more mother-friendly occupations that may lead to a lower observed labor income over their life cycle. In Denmark a more mother-friendly sector is the public sector, and Danish women with the higher earnings penalty of children self-select into the public sector, which pays lower wages but has a less severe penalty of child leave [20].

## Conclusion

We analyze the relationship between the age at first birth and the lifetime labor income of mothers using Danish administrative register data from 1995–2009. Our results show that the short-run loss in labor income, defined as the difference in percentages between the income earned two years prior to AFB and income earned at AFB, range from 37% to 65% for college women; the range was 40% to 53% for non-college women. We also find a monotonic relationship between the lifetime income differentials and AFB. Our results suggest a trade-off between the decision of having a child during the earliest stages of women’s careers and lifetime labor income with clear lower lifetime labor income for AFB<25. The higher labor income associated with having a first child later in life at least until age 31 are clear for both college and non-college women. Our findings highlight the importance of considering jointly fertility and career decisions when analyzing women labor market outcomes.
